# The correlation between lung ultrasound scores and outcomes of high-flow nasal cannula therapy in infants with severe pneumonia

**DOI:** 10.1186/s12887-024-04522-7

**Published:** 2024-01-16

**Authors:** Li-Ling Zheng, Rou Chen, Chan-Hua Zheng, Xiao-Juan Dai, Wei-Da Zheng, Jia-Xiang Zhang

**Affiliations:** Department of Pediatric Intensive Care Unit, Zhangzhou Affiliated Hospital, Fujian Medical University, 59 Shengli West Road, Xiangcheng District, Zhangzhou, China

**Keywords:** High-flow nasal cannula, Severe pediatric pneumonia, Pulmonary ultrasound, Oxygenation index

## Abstract

**Objective:**

The study aimed to explore the effectiveness of bedside lung ultrasound (LUS) combined with the PaO_2_/FiO_2_ (P/F) ratio in evaluating the outcomes of high-flow nasal cannula (HFNC) therapy in infants with severe pneumonia.

**Methods:**

This retrospective study analyzed the clinical data of 150 infants diagnosed with severe pneumonia and treated with HFNC therapy at our hospital from January 2021 to December 2021. These patients were divided into two groups based on their treatment outcomes: the HFNC success group (*n* = 112) and the HFNC failure group (*n* = 38). LUS was utilized to evaluate the patients’ lung conditions, and blood gas results were recorded for both groups upon admission and after 12 h of HFNC therapy.

**Results:**

At admission, no significant differences were observed between the two groups in terms of age, gender, respiratory rate, partial pressure of oxygen, and partial pressure of carbon dioxide. However, the P/F ratios at admission and after 12 h of HFNC therapy were significantly lower in the HFNC failure group (193.08 ± 49.14, 228.63 ± 80.17, respectively) compared to the HFNC success group (248.51 ± 64.44, 288.93 ± 57.17, respectively) (*p* < 0.05). Likewise, LUS scores at admission and after 12 h were significantly higher in the failure group (18.42 ± 5.3, 18.03 ± 5.36, respectively) than in the success group (15.09 ± 4.66, 10.71 ± 3.78, respectively) (*p* < 0.05). Notably, in the success group, both P/F ratios and LUS scores showed significant improvement after 12 h of HFNC therapy, a trend not observed in the failure group. Multivariate regression analysis indicated that lower P/F ratios and higher LUS scores at admission and after 12 h were predictive of a greater risk of HFNC failure. ROC analysis demonstrated that an LUS score > 20.5 at admission predicted HFNC therapy failure with an AUC of 0.695, a sensitivity of 44.7%, and a specificity of 91.1%. A LUS score > 15.5 after 12 h of HFNC therapy had an AUC of 0.874, with 65.8% sensitivity and 89.3% specificity. An admission P/F ratio < 225.5 predicted HFNC therapy failure with an AUC of 0.739, 60.7% sensitivity, and 71.1% specificity, while a P/F ratio < 256.5 after 12 h of HFNC therapy had an AUC of 0.811, 74.1% sensitivity, and 73.7% specificity.

**Conclusion:**

Decreased LUS scores and increased P/F ratio demonstrate a strong correlation with successful HFNC treatment outcomes in infants with severe pneumonia. These findings may provide valuable support for clinicians in managing such cases.

## Introduction

 Severe pneumonia represents a frequent emergency in infant and pediatric care. It can affect multiple systems, presenting with symptoms such as dyspnea, respiratory distress, cyanosis, and even systemic poisoning symptoms, often leading to fatalities in children under five years old. Most infants with severe pneumonia experience hypoxemia and dyspnea, necessitating effective mechanical respiratory support. A substantial body of research indicates that high-flow nasal cannula (HFNC) therapy can reduce intubation and mortality rates in infants and children with an oxygenation index (PaO_2_/FiO_2_ ratio, P/F ratio) below 200 mmHg [1, 2]. Recently, HFNC therapy has gained increasing prominence in pediatric intensive care units (PICUs), demonstrating effectiveness in improving oxygenation and reducing the need for tracheal intubation in infant and pediatric patients. A systematic review and network meta-analysis revealed that continuous positive airway pressure (CPAP), HFNC therapy, and bilevel positive airway pressure (BiPAP) resulted in lower extubation and treatment failure rates compared to conventional oxygen therapy (COT) [1]. However, HFNC therapy is not without its failures. Factors predictive of HFNC therapy failure include underlying chronic disease, low diastolic blood pressure, high respiratory and heart rates, and elevated initial PaCO_2_ levels [2]. 

Bedside lung ultrasound (LUS) is a noninvasive tool that can dynamically monitor lung changes in real time. Studies in low- and middle-income countries suggest that LUS may be useful in diagnosing and treating severe pneumonia in infants and children [3]. Another study indicated that novice and experienced sonographers could reduce chest X-ray usage by 30.0% and 60.6%, respectively, when using LUS, concluding that substituting LUS for chest X-ray in evaluating infants with suspected pneumonia is feasible and safe, without missing any pneumonia cases or increasing adverse event rates [4]. 

The oxygenation index is a valuable metric for assessing the extent of hypoxemia in infants and can more accurately quantify the influence of pressure support during oxygenation. By combining LUS with the P/F ratio from blood gas analysis, the monitoring of patient breathing and lung conditions can be more effectively conducted. Therefore, this study aims to investigate whether the LUS score combined with the P/F ratio can effectively evaluate HFNC treatment outcomes in infants with severe pneumonia.

## Materials and methods

### Study population and data collection

This is a retrospective study. In this study, we focused on infants aged 29 days to 1 year who were diagnosed with severe pneumonia and received HFNC therapy at our hospital between January 2021 and December 2021. The criteria for severe pediatric pneumonia were based on the definitions provided in the ninth edition of Pediatrics. Exclusions were made for patients with congenital heart disease or severe pneumonia secondary to conditions like brain tumors, those who required HFNC therapy due to intolerance to nasal catheters at admission, and cases where treatment was discontinued or patients were discharged or deceased at the family’s request. All participants underwent chest X-rays or CT scans during their hospital stay, and we collected collected blood gas results, ultrasound images and scores at admission and 12 h after HFNC treatment. This study adhered to ethical standards and was approved by the Hospital Medical Ethics Committee.

### Study group

Patients in this study were categorized into two groups based on their response to HFNC therapy: the HFNC success group and the HFNC failure group. Those who were successfully weaned off HFNC therapy were placed in the success group (*n* = 112), while patients whose conditions worsened necessitating invasive mechanical ventilation were classified in the failure group (*n* = 38). Both groups underwent identical diagnostic examinations and received comparable adjunctive treatments.

### HFNC treatment

The HFNC therapy equipment used in this study included air and oxygen mixing devices, an MR850 humidifier equipped with an RT329 respiratory pipeline, and specialized nasal catheters. The parameters for the HFNC therapy were set as follows: the initial flow rate was maintained between 1 and 2 L per minute (l/min/kg), and the fraction of inspired oxygen (FiO_2_) was set between 40 and 50%. There was dynamic monitoring of blood oxygen saturation, blood gases, and oxygenation status, with timely adjustments made to FiO_2_ as needed. The temperature of the delivered air was regulated to range from 31 to 37 °C.

### Bedside LUS method

#### Observation indicators and methods

**Fig. 1:**
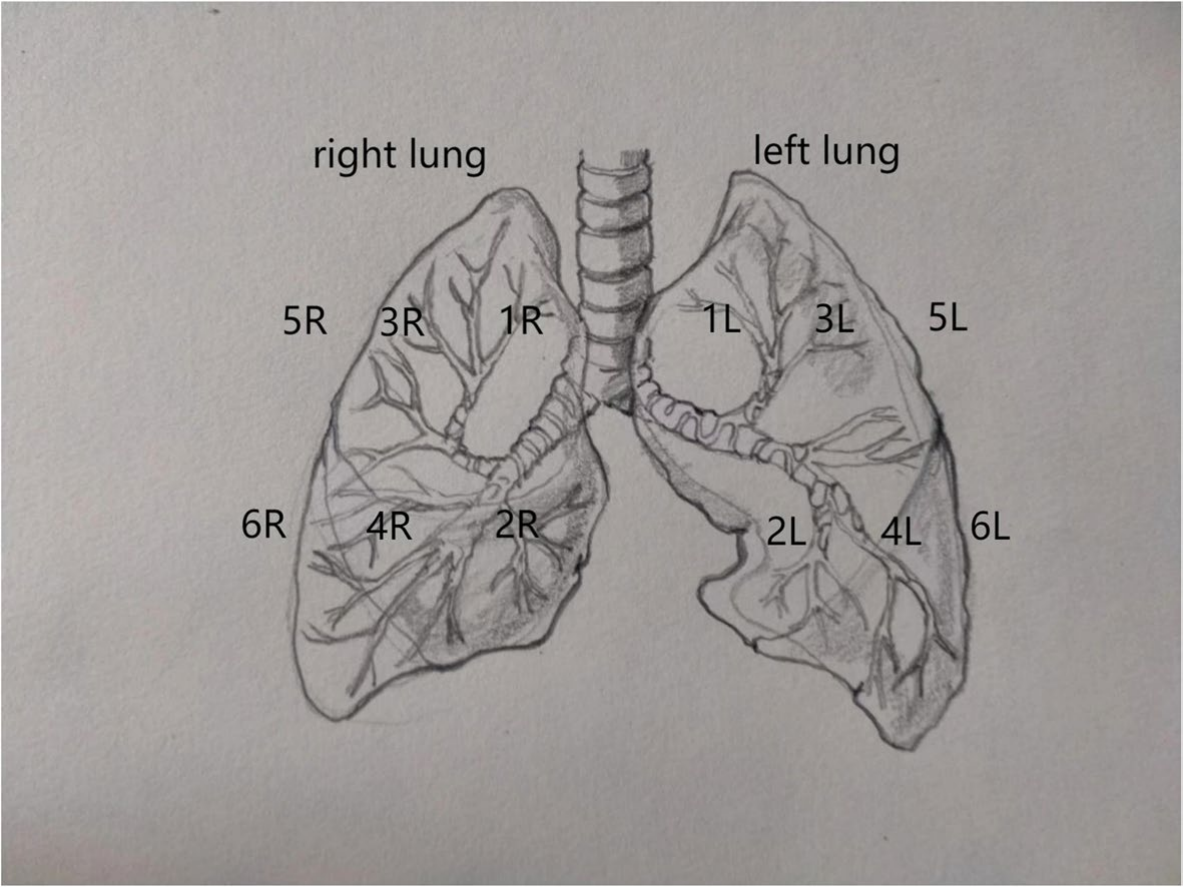
Pulmonary ultrasound 12 zone diagram. Left lung zones: L1, left upper anterior; L2, left lower anterior; L3, left upper lateral; L4, left lower lateral; L5, left upper posterior; L6, left lower posterior. R refers to the corresponding area on the right side

Bedside LUS was primarily conducted by the first-line attending physicians in the PICU, who were certified in bedside ultrasound operation by the hospital’s ultrasound department. The VenueGo ultrasound machine equipped with the Lung Tool and Lung Scoring toolkit was utilized for these examinations. Point-of-Care Ultrasound (PoCUS) was performed using a linear array probe (GE) with a frequency range of 4–12 Hz, encompassing both transverse and longitudinal scans. The scanning area was based on the protocol described in Manivel V’s research on the application of lung ultrasound in COVID-19 [5]. (Fig. 1) Preliminary clinical assessments and image interpretations were conducted immediately, with subsequent reevaluation by the ultrasound team. LUS scores for both groups were recorded at admission and after 12 h of HFNC therapy. Concurrently, blood gas oxygenation, changes in the P/F ratio, heart rate, respiration, and basic data were also documented.

### LUS scheme

**Fig. 2:**
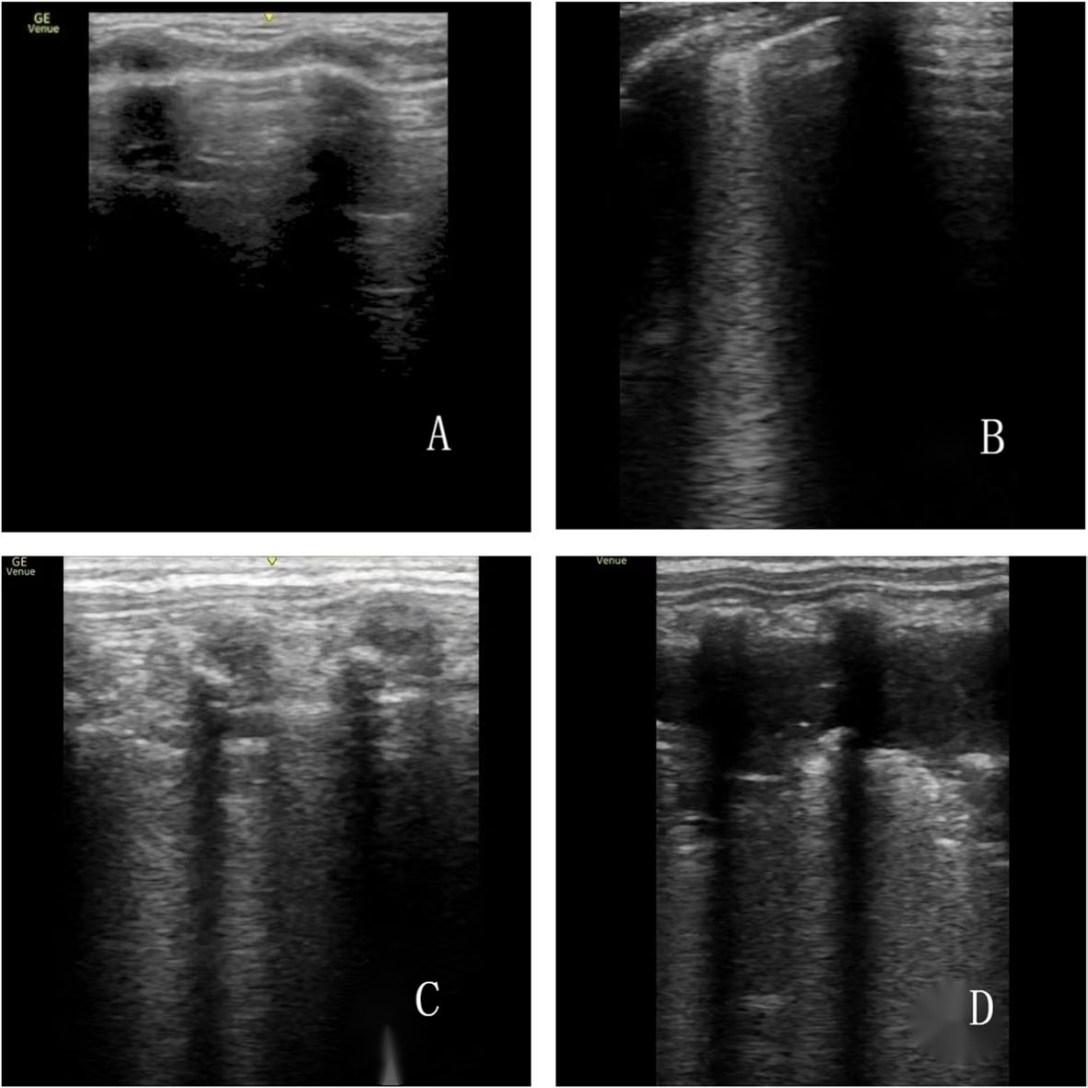
Ultrasound images under different lung ultrasound scores. **A** is 0 score ultrasound image, indicating normal signs; **B** is 1 score ultrasound image, indicating a small amount of B line; **C** is 2 scores ultrasound image, indicating diffuse fusion of line B; **D** is 3 scores ultrasound image, indicating extensive lung consolidation

In our LUS scoring system, each of the 12 bilateral lung zones could receive a score from 0 to 3, culminating in a maximum possible total score of 36. The overall LUS score was a cumulative total of the individual scores from these 12 zones, with higher scores indicating poorer lung ventilation. The scoring criteria were as follows: a score of 0 was assigned for an A-line pattern, characterized by the exclusive presence of A lines; a score of 1 denoted a B-line pattern, identified by the presence of three or more well-spaced B lines; a score of 2 was given for a severe B-line pattern, marked by crowded and coalescent B lines, with or without subpleural space consolidations; and a score of 3 indicated extensive consolidations (as illustrated in Fig. 2). In this context, A lines represent the pleural reflection due to ultrasound waves diffusing through an air-filled lung, whereas B lines signify fluid in the interstitium, which may also involve the alveolar space if they are confluent [6]. 

### Therapeutic method

All infants diagnosed with severe pneumonia were treated with HFNC therapy alongside other standard treatments for respiratory infections. We continuously monitored percutaneous oxygen saturation (SpO_2_), blood gases, and respiratory function. Patients were classified into the HFNC success group if, post-HFNC therapy, they demonstrated overall clinical improvement, had an P/F ratio greater than 300, and were able to reduce FiO_2_ to 30% without experiencing hypoxia upon HFNC withdrawal. Additionally, successful cases were those who maintained adequate ventilation with a SpO_2_ exceeding 95%. Conversely, patients were assigned to the HFNC failure group if, after HFNC therapy, they required FiO_2_ levels above 60%, exhibited disease progression with decreased levels of consciousness, were unable to maintain blood oxygen saturation above 90%, had a partial pressure of carbon dioxide exceeding 60 mmHg, or necessitated escalation to invasive mechanical ventilation.

### Statistical analysis

In our analysis, data following a normal distribution were expressed as means and standard deviations, and comparisons between the two groups were conducted using an independent sample t-test. Count data were presented as frequencies and percentages, with the chi-square test applied for comparative analysis. We employed multivariate logistic regression to evaluate the predictive value of the LUS score, incorporating it along with other covariates (selected through a stepwise process) as independent variables, and HFNC therapy failure as the dependent variable. The sensitivity (Se), specificity (Sp), and the area under the receiver operating characteristic (ROC) curve (AUC) were calculated to assess the diagnostic performance. Data processing and analysis were conducted using SPSS software version 26.0, and a *p*-value of less than 0.05 was considered indicative of statistical significance.

## Results

### Patient characteristics

In this study, a total of 150 infants were included. The HFNC success group comprised 112 infants, with 72 males and 40 females, and an average age of 5.25 ± 4.13 months. The HFNC failure group consisted of 38 infants, including 25 males and 13 females, with a mean age of 4.95 ± 4.02 months. The average respiratory rate was 52.46 ± 7.16 breaths per minute in the success group and 54.42 ± 8.52 breaths per minute in the failure group. No significant statistical differences were observed in age, gender, or respiratory rate at admission between the two groups. The mean heart rate at admission was 162.27 ± 14.61 beats per minute in the success group and 167.71 ± 22.77 beats per minute in the failure group, showing significant difference (*p* < 0.05). After 12 h of HFNC therapy, there was no significant difference in heart rate between the two groups. (Table 1) The length of stay in the PICU was notably longer for the failure group (11.16 ± 4.52 days) compared to the success group (6.25 ± 3.32 days) (*p* < 0.05).

**Table 1:** Comparison of general conditions between the two groups

	Success group (*n* = 112)	Failure group (*n* = 38)	t value	* p* value
Age at admission (month)	5.25 ± 4.13	4.95 ± 4.02	0.724	0.396
Gender at admission (male/female)	72/40	25/13	0.042	0.838
Respiratory rate admission (time/min)	52.46 ± 7.15	54.42 ± 8.52	0.084	0.773
Respiratory rate after 12 h of HFNC therapy (time/min)	38.08 ± 7.82	42.53 ± 8.20	0.283	0.596
Heart rate admission (time/min)	162.27 ± 14.61	167.71 ± 22.77	14.87	0.00
Heart rate after 12 h of HFNC therapy (time/min)	138.12 ± 20.54	142.18 ± 17.45	1.377	0.243
Length of stay in the PICU (days)	6.25 ± 3.32	11.16 ± 4.52	4.74	0.031

### Blood gas index, P/F ratios, and LUS score

At admission and after 12 h of HFNC therapy, there was no notable difference in either oxygen partial pressure or carbon dioxide partial pressure between the groups. All infants underwent a LUS examination upon admission, revealing varying degrees of exudation in most cases, and some showed fragmented signs. Follow-up LUS was performed after 12 h of HFNC therapy to assess any increase in exudation, worsening lung consolidation, or the presence of extensive lung consolidation shadows. Compared to the initial assessment at admission, the P/F ratios and LUS scores significantly improved in the success group following 12 h of HFNC therapy, but no similar improvement was observed in the failure group.

The P/F ratios at admission and after 12 h of HFNC therapy showed significant differences between the groups. In the HFNC failure group, the P/F ratios were markedly lower (193.08 ± 49.14 at admission and 228.63 ± 80.17 after 12 h) compared to the success group (248.51 ± 64.44 at admission and 288.93 ± 57.17 after 12 h) (*p* < 0.05). Similarly, LUS scores were significantly higher in the failure group both at admission (18.42 ± 5.3) and after 12 h of therapy (18.03 ± 5.36), as opposed to the success group, which showed scores of 15.09 ± 4.66 at admission and 10.71 ± 3.78 after 12 h (*p* < 0.05) (Table 2).

**Table 2:** Comparison of blood gas indexes and P/F value and LUS scores before and after treatment in the two groups

	Success group (*n* = 112)	Failure group (*n* = 38)	t value	* p* value
**PO**_**2**_ ** at admission** **(mmHg)**	86.82 ± 30.36	81.13 ± 29.33	0.168	0.682
**PCO**_**2**_ ** at admission (mmHg)**	40.61 ± 9.54	44.84 ± 12.43	3.576	0.061
**PO**_**2**_ ** after 12 h of HFNC therapy (mmHg)**	102.81 ± 38.28	99.47 ± 26.67	3.1	0.08
**PCO**_**2**_ ** after 12 h of HFNC therapy** **(mmHg)**	38.29 ± 8.93	38.58 ± 8.73	0.393	0.532
**P/F at admission**	248.51 ± 66.44	193.08 ± 49.14	4.554	0.034
**P/F after 12 h of HFNC therapy**	288.93 ± 57.17	228.63 ± 80.17	10.054	0.002
**LUS scores at admission**	15.09 ± 4.66	18.71 ± 5.46	4.286	0.04
**LUS scores after 12 h of HFNC therapy**	10.71 ± 3.77	18.03 ± 5.36	5.877	0.017

### Multivariate logistic regression analysis

Following variable selection, the P/F ratios from blood gas analysis and LUS scores at admission, as well as after 12 h of HFNC therapy, were included as independent variables in our analysis. The findings indicated that higher LUS scores and lower P/F ratios, both at admission and after 12 h of HFNC therapy, were associated with an increased risk of HFNC withdrawal failure (Table 3).

**Table 3:** Multivariate logistic regression analysis

Variation	B	SE	Walds	* p*	OR	95% CI
**P/F at admission**	0.012	0.009	1.928	0.165	1.012	0.995 ~ 1.030
**P/F after 12 h of HFNC therapy**	0.018	0.006	8.059	0.005	1.018	1.006 ~ 1.031
**LUS at admission**	0.571	0.144	15.67	0.000	1.769	1.334 ~ 2.347
**LUS after 12 h of HFNC therapy**	-0.860	0.175	24.036	0.000	0.423	0.300 ~ 0.597

### ROC analysis

**Fig. 3:**
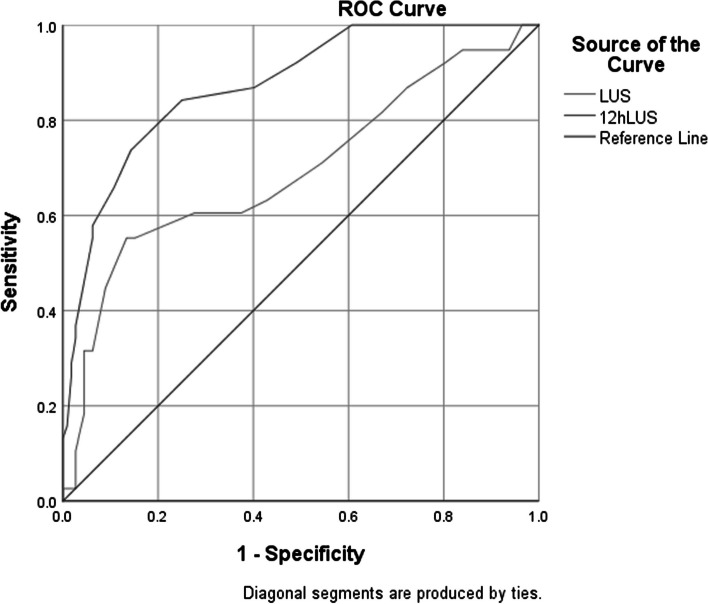
ROC curve of successful withdrawal by lung ultrasound score in predicting the outcome of HFNC

**Fig. 4:**
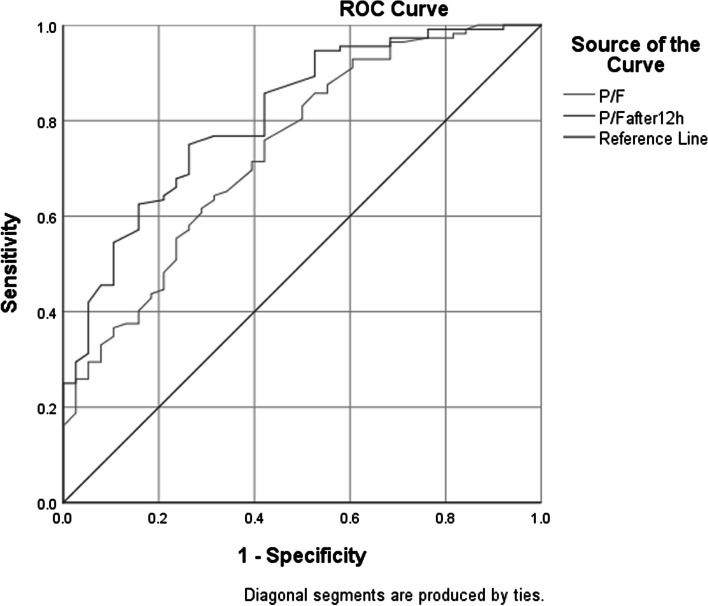
ROC curve of successful withdrawal by P/F value in predicting the outcome of HFNC

ROC analysis was utilized to assess the predictive capability of the LUS score both at admission and after 12 h of HFNC therapy. An LUS score above 20.5 at admission predicted HFNC failure with a sensitivity of 44.7%, specificity of 91.1%, and an AUC of 0.695 (95% confidence interval [CI]: 0.588–0.801). When the LUS score exceeded 15.5 after 12 h of HFNC therapy, it indicated HFNC failure with a sensitivity of 65.8%, specificity of 89.3%, and an AUC of 0.874 (95% CI: 0.811–0.936) (Fig. 3). A P/F ratio less than 225.5 at admission was predictive of HFNC failure with a sensitivity of 60.7%, specificity of 71.1%, and an AUC of 0.739 (95% CI: 0.648–0.831). Similarly, a P/F ratio below 256.5 after 12 h of therapy predicted failure with a sensitivity of 74.1%, specificity of 73.7%, and an AUC of 0.811 (95% CI: 0.735–0.888) (Fig. 4).

## Discussion

HFNC oxygen therapy has been demonstrated to be effective in treating mild to moderate acute hypoxic respiratory failure in infants and children [7, 8]. However, despite HFNC therapy, some patients still progress to requiring invasive ventilation. The early identification of predictors for HFNC therapy failure is vital, given the diverse etiologies of pediatric respiratory failure. Our findings indicate that a decreased P/F ratio and increased LUS scores at admission and 12 h post-HFNC initiation are associated with a higher risk of HFNC therapy failure. ROC analysis revealed that a P/F ratio ≤ 222.5 at admission had a specificity of 71.1% in predicting HFNC therapy failure, while a P/F ratio ≤ 256.5 after 12 h of HFNC therapy had a specificity of 73.7%. Additionally, an LUS score > 20.5 at admission and > 15.5 after 12 h of HFNC therapy demonstrated specificities of 91.1% and 89.3%, respectively, in predicting HFNC therapy failure. These findings suggest that combining P/F ratio and LUS score assessments could effectively predict outcomes of HFNC therapy.

LUS is highly sensitive in detecting lung consolidation, making it a suitable alternative to chest radiography in PICU. LUS estimates lung aeration through semi-quantitative scoring of ultrasound patterns, as supported by studies [9, 10]. It quantifies the loss of ventilated lung areas, thus accurately depicting the nonlinear relationship between ventilation and lung area. Higher LUS scores indicate more impaired ventilation, correlating with the severity of the disease [11]. Research has demonstrated that emergency LUS effectively identifies the causes of pediatric respiratory failure [12]. Enhanced LUS scoring has further refined the quantification of lung consolidation, enabling accurate, real-time assessments of ventilation changes and the extent of lung involvement [13]. Therefore, LUS monitoring is crucial in guiding decisions on the timing of HFNC withdrawal and predicting outcomes, showing its potential as a specific predictor of successful HFNC withdrawal in severe pneumonia cases among infants and children.

The advancement of LUS technology has significantly enhanced the diagnosis and severity assessment of lung diseases, both domestically and internationally [14]. LUS is increasingly recognized for its ability to predict ventilatory support needs and assist in clinical decision-making in adult patients, although studies in infantile and pediatric populations remain limited [15]. In 2020, expert consensus underscored the importance of LUS in managing pediatric pneumonia and bronchiolitis, offering a radiation-free alternative to chest CT [16]. Elevated LUS scores at admission have been linked to increased care intensity and mortality among COVID-19 patients [17]. The ongoing COVID-19 pandemic and related public health measures have likely contributed to a reduced circulation of other respiratory viruses compared to pre-pandemic levels [18]. Despite a decrease in severe pneumonia emergencies, the need for early respiratory assessment remains paramount. A study involving 103 pediatric admissions for respiratory distress found that LUS scores above 12 had a high specificity (positive likelihood ratio of 8.74) for diagnosis [19]. Our study aligns with these findings, showing that LUS scores correlate with patient prognosis and severity, and appear feasible for predicting HFNC therapy failure. The correlation between LUS scores and the P/F ratio was consistent regardless of ultrasound patterns. LUS has been effective in diagnosing and stratifying COVID-19 pneumonia prognosis [20]. Posttreatment increases in LUS scores have been observed, predicting ongoing dyspnea in COVID-19 cases [21]. These results are in line with our observations that infantile LUS scores are effectively associated with HFNC therapy outcomes in infants with severe pneumonia, thereby complementing oxygenation metrics.

Multivariate regression analysis in our study revealed that both P/F ratios and LUS scores at admission and after 12 h of HFNC therapy were associated with the risk of HFNC failure. The P/F ratio is known to be a prognostic marker for acute hypoxemic respiratory failure, underscoring its predictive value for HFNC therapy success, as our findings support [22]. Patients with more severe pneumonia typically exhibit lower P/F ratios and higher LUS scores, suggesting a relationship with the severity of pneumonia [23]. Our results confirmed this negative association between LUS scores and the P/F ratio both at admission and after 12 h of HFNC therapy, indicating an LUS-oxygenation abnormality link primarily in acute disease presentations. Factors like progressive fluid overload and atelectasis may affect LUS changes throughout the disease course. This aligns with our observation that higher LUS scores and lower P/F ratios are indicative of HFNC treatment failure [23]. A separate ICU cohort study reported increased mortality rates among patients with higher LUS scores and lower P/F ratios, suggesting that LUS scores can independently predict mortality [24]. Thus, the combined use of effective LUS and P/F ratio assessments enhances the accuracy of predicting overall HFNC therapy outcomes.

Bedside LUS is an ideal, noninvasive, real-time monitoring technique for critically ill pediatric patients, with its B lines showing significant correlation with pulmonary edema and fibrosis [25]. Additionally, bedside blood gas analysis is a readily available resource. Therefore, combining LUS scores with P/F ratios might better predict outcomes of HFNC therapy in infants with severe pneumonia in the future.

### Limitation

Our study, however, is not without limitations. The absence of routine pediatric chest CT, primarily due to radiation concerns, and the variability in examination timing posed challenges in predicting HFNC therapy failure. More frequent LUS assessments at intervals such as 6, 12, and 24 h post-treatment initiation could potentially enhance the predictive value for HFNC failure. We did not include the correlation between the time point of HFNC treatment failure and LUS. Being a retrospective study, it is not possible to provide further evidence on the temporal correlation between LUS results and HFNC treatment failure, necessitating future prospective studies. Additionally, the single-center retrospective design, the relatively small sample size, and the limited range of indicators may impact the accuracy of our findings.

## Conclusion

Decreased LUS scores and increased P/F ratios demonstrate a strong correlation with successful HFNC treatment outcomes in infants with severe pneumonia. This correlation may provide valuable support in the treatment and management of these patients.

## Data Availability

The datasets used and analyzed during the current study are available from the corresponding author on reasonable request.
